# Usefulness of Endoscopic Retrograde Cholangiopancreatography (ERCP)-Related Procedures for Gallstone Pancreatitis

**DOI:** 10.7759/cureus.67133

**Published:** 2024-08-18

**Authors:** Yuji Sakai, Toshio Tsuyuguchi, Junichiro Kumagai, Hiroshi Ohyama, Taka-aki Nakada, Masayuki Ohtsuka, Naoya Kato

**Affiliations:** 1 Department of Gastroenterology, Sakai Clinic, Kimitsu, JPN; 2 Department of Gastroenterology, Graduate School of Medicine, Chiba University, Chiba, JPN; 3 Department of Gastroenterology, Chiba Prefectural Sawara Hospital, Sawara, JPN; 4 Department of Gastroenterology, Kimitsu Central Hospital, Kisarazu, JPN; 5 Department of Emergency and Critical Care Medicine, Chiba University, Chiba, JPN; 6 Department of General Surgery, Graduate School of Medicine, Chiba University, Chiba, JPN

**Keywords:** acute pancreatitis, gall stone disease (gsd), endoscopic sphincterotomy, endoscopy ercp, gallstone pancreatitis

## Abstract

Introduction: Endoscopic retrograde cholangiopancreatography (ERCP) and endoscopic sphincterotomy (EST) are said to be useful procedures for gallstone pancreatitis. However, there have been few reports on a large number of patients to whom they were used. The clinical usefulness of ERCP and EST is herein examined retrospectively.

Methods: ERCP and EST were performed to evaluate their clinical usefulness and safety in 108 patients who had gallbladder stones from December 1985 to October 2017 and were diagnosed as having gallstone pancreatitis. Of 108 patients, 83 were mild, and 25 were severe. Following the procedures, clinical courses were observed for three years in 108 patients who underwent the treatments.

Results: Cholangiogram was successfully conducted in 108 patients. Bile duct stones were noted in 90 patients, and the stones were removed after EST. Of 18 patients who did not show bile duct stone in cholangiogram, 13 patients underwent EST, while five patients taking anticoagulants completed procedures only with cholangiogram. Accidental symptom was hemorrhage in three patients (2.7%; 3/108), but it was mild and conservatively resolving. During the three-year observation period, acute cholangitis was noted in three patients (2.7%; 3/108), but no relapsing pancreatitis was noted.

Conclusions: It was suggested that ERCP and EST could be useful therapies for gallstone pancreatitis.

## Introduction

Acute pancreatitis is diagnosed and its causes are searched by taking medical history and blood/urine samples from patients and through diagnostic imaging. Treatment of acute pancreatitis is based on fasting, adequate fluid replacement, and sufficient removal of pain [1]. Depending on its cause and severity, however, the treatment plans may vary. Caution is required, as there are cases where an unfortunate outcome occurs if appropriate treatment is not administered. Gallbladder stones are the second most common cause of acute pancreatitis after alcohol, and it has been reported that they account for around 25% of cases of acute pancreatitis [1]. If the cause of acute pancreatitis is alcohol or unknown, the abovementioned treatment is the basis. But, if the cause is gallstone, endoscopic retrograde cholangiopancreatography (ERCP)-related procedures need to be taken into consideration [1,2]. ERCP-related procedures for gallstone pancreatitis are said to be useful when a blocked duct is suspected [3-6]. There is, however, a paucity of studies on a large number of patients, and studies with a sample size exceeding 100 cases are extremely rare [4]. Because ERCP-related procedures have the potential to worsen the pathological condition of pancreatitis, we believe that short-term studies of a small number of cases are insufficient. This study sheds light on 108 patients with gallstone pancreatitis we have experienced in the past 30 years or so.

## Materials and methods

We studied 108 patients who had gallbladder stones from December 1985 to October 2017 and were diagnosed as having gallstone pancreatitis at our hospital. Excluding other pancreatic diseases and acute abdomen, diagnosis of acute pancreatitis was made when two of the following three items were met: (a) acute abdominal pain attack and tenderness on the upper abdomen, (b) elevation of pancreatic enzyme in blood and urine, and (c) abnormal findings on ultrasonography, CT, or MRI in the pancreas, associated with acute pancreatitis [7]. Diagnosis of gallstone pancreatitis was made when a common bile duct stone was noted on images or when there were abnormal findings in more than three of the following five blood test items: bilirubin, alkaline phosphatase (ALP), gamma-glutamyl transpeptidase (γ-GTP), alanine transaminase (ALT), and ALT/aspartate transferase (AST) ratio [8]. Glasgow score was used to rate severity [9]. Severity was scored on the basis of the Glasgow score after hospitalization. When a diagnosis of gallstone pancreatitis was made, protease inhibitors and antibiotics were administered, depending on the conditions of the patients. Prior to ERCP-related procedures, patients and their families were given a full explanation of the clinical study. After obtaining the informed consent, we performed the procedures. ERCP-related procedures were performed when our hospital diagnosed gallstone pancreatitis. Expert physicians experienced in more than 1000 cases of ERCP-related procedures performed the operations. Before ERCP, all patients were given a standard premedication consisting of intravenous administration of midazolam (3-10 mg) or diazepam (2-5 mg), and the dose depended on age and tolerance. Butylscopolammonium bromide or glucagon was used to suppress peristaltic activities of the gastrointestinal tract. During ERCP, more than two physicians and one or more nurses monitored the patient, using an ECG monitor and a pulse oximeter. A therapeutic duodenoscope was used. We used a therapeutic duodenoscope (JF200, JF240, or JF260V, Olympus, Tokyo, Japan). For cannulation, a contrast method using a normal catheter was used (PR-104Q, R110Q-1, or PR233Q, Olympus, Tokyo, Japan). A 0.025-inch or 0.035-inch guidewire (Jagwire, Boston Scientific, Natick, Massachusetts, United States; VisiGlide, Olympus, Tokyo, Japan) was used. Endoscopic sphincterotomy (EST) was performed using a sphincterotome so as to make the opening of the bile duct separate from that of the pancreatic duct (KD-10Q, Clever-Cut3V, or KD-P0610Q, Olympus, Tokyo, Japan). EST was conducted using a single electrosurgical current generator (PSD-20, Olympus, Tokyo, Japan) at a power of 25 watts. Instruments used and procedures were selected at the discretion of operating physicians. The Cotton Classification was used to evaluate accidental symptoms following ERCP-related procedures [10]. After the evaluation, clinical courses of 108 patients were observed for three years. This study was approved by the Ethical Committee of Chiba University Hospital (approval number: HK202403-13). This study is retrospective.

## Results

Table 1 shows the patient background: 55 men and 53 women with a mean (±standard deviation (SD)) age of 66.670±11.136 (years old). The severity assessment of acute pancreatitis resulted in 83 mild cases (scores 0-2) and 25 severe cases (scores 3-8), and the average Glasgow score (±SD) was 2.196±2.090.

**Table 1:** Background of patients SD: standard deviation

	n=108
Age, (±SD)	66.670±11.136
Sex, male, (%)	55 (50.9)
Female, (%)	53 (49.1)
Pancreatitis	
Glasgow score, (±SD)	2.196±2.090
Grade	
Mild, (%)	83 (76.9)
Severe, (%)	25 (23.1)

ERCP was performed on 108 patients. The results are shown in Table 2.

**Table 2:** Results of ERCP ERCP: endoscopic retrograde cholangiopancreatography; EST: endoscopic sphincterotomy

	n=108
Bile duct cannulation, (%)	108 (100)
Stone, (%)	90 (83.3)
Bile duct stone, (%)	78 (72.2)
Incarcerated stone, (%)	12 (11.1)
Number of stones, (±SD)	1.166±2.090
Size (mm), (±SD)	5.223±6.888
EST, (%)	103 (95.4)
Number of ERCP performed, (±SD)	1.123±1.225
Accidental symptoms, (%)	
Hemorrhage, (%)	3 (2.8)

Cholangiogram was successfully conducted in 108 patients. Stones were noted in 90 patients (83.3%), and of 90, 12 patients (11.1%) showed incarcerated stones in the duodenal papilla. The mean (±SD) numbers of stones were 1.166±2.090, and the mean (±SD) stone diameters were 5.223±6.888 mm. Stones were removed after performing EST. Of 18 patients (16.7%) who did not show bile duct stone in cholangiogram, 13 patients underwent EST, while five patients taking anticoagulants completed procedures only with cholangiogram. When a single ERCP could not remove stones, a plastic stent was inserted, and stones were removed at a later date. The mean (±SD) number of ERCP operations was 1.123±1.225. Stones were successfully removed in all 90 patients. Following ERCP, 12 patients (11.1%) of all severe cases were managed in the intensive care unit. As post-ERCP complications, hemorrhage was noted in three patients (2.8%), but it was mild and conservatively resolving. There was no death case during the treatment. The results of pancreatitis treatment are shown in Table 3.

**Table 3:** Treatment results of pancreatitis EUS: endoscopic ultrasonography

	n=108
Life-saving, (%)	108 (100%)
Complications, (%)	14 (13%)
Pancreatic pseudocyst, (%)	6 (5.6%)
Spontaneous absorption, (%)	2 (1.9%)
EUS-guided drainage, (%)	4 (3.7%)
Walled-off necrosis, (%)	8 (7.4%)
EUS-guided necrosectomy, (%)	8 (7.4%)
Pancreatitis relapsing, (%)	0 (0%)

In all 108 patients, pancreatitis improved. After the improvement of pancreatitis, pancreatic pseudocyst was noted in six patients (5.6%), and walled-off necrosis was noted in eight patients (7.4%). As for patients with pancreatic pseudocyst, spontaneous absorption was noted in two patients (1.9%) during the clinical course observation, but spontaneous absorption did not occur in four patients, whose pancreatic pseudocysts disappeared after endoscopic ultrasonography (EUS)-guided drainage. As for eight patients with walled-off necrosis, necrotic cell debris was successfully removed by endoscopic necrosectomy four weeks after the onset of pancreatitis, and all eight patients recovered. During the three-year observation period, 36 patients (33.3%) underwent cholecystectomy (17 underwent open cholecystectomy, 19 underwent laparoscopic cholecystectomy). No relapsing of acute pancreatitis was noted during the observation period. However, acute cholangitis attributable to a fall of gallbladder stone was noted in three patients (2.8%), and their stones were removed by ERCP. No accidental symptom relating to the procedure was noted. In patients who underwent cholecystectomy, no acute cholangitis was noted during the observation period.

## Discussion

Treatment results of acute pancreatitis have improved owing to the recent progress in diagnostic imaging, intensive treatments including transfusion/nutrition management, the introduction of continuous hemodiafiltration as a special treatment in severe cases [11], and reports of usefulness of new treatment drugs including recombinant human soluble thrombomodulin [12]. Yet, we still see death cases [11,12]. Treatment plans vary depending on the cause and severity of acute pancreatitis. Gallstone pancreatitis is caused by gallstones, which block the common channel and pancreatic duct and raise the intraductal pressure of the pancreas. Better results were reported in randomized controlled trials when ERCP and EST were performed to clear biliary obstruction, rather than conservative treatments [3-6]. If gallstone pancreatitis suggests biliary obstruction, an endoscopic procedure is recommended [1]. The problem here is whether or not biliary obstruction is present. This time, we performed ERCP since gastrointestinal obstruction was suspected; however, 16.7% (18/108) of the patients had no bile duct stone. For gallstone pancreatitis, the causative stone is most likely to be small. Since the cystic duct has a small diameter, small stones are likely to fall from the gallbladder to the common bile duct. If stones are bigger, obstruction is likely to occur at a lower bile duct level. For this reason, it is important to use diagnostic imaging to detect small stones. In some cases, spontaneous passage of bile duct stones occurs [13-15]. Since ERCP may worsen pancreatitis due to pancreatic duct imaging or the incorrect insertion of a guidewire into the pancreatic duct, careful consideration must be given to the suitability of ERCP. What is needed here is a high detection rate of bile duct stones. CT is very useful in diagnosing acute pancreatitis. Despite its high detectability of bile duct stones reported to date [16], CT is not optimal when visualizing bile duct stones with a little amount of calcium component. Moreover, its detection sensitivity of bile duct stone is as low as 40-53% [8,17]. High detectability of bile duct stone has been reported with magnetic resonance cholangiopancreatography (MRCP) [13-18]. However, in case the bile duct has a large diameter, patients have ascites retention, or a bile duct stone is located near the duodenal papilla, small stones may not be detected because bile is less likely to be anatomically accumulated [18]. Hence, some argue that EUS should be used to detect bile duct stones. It has been reported that EUS itself has no chance of triggering pancreatitis, that it does not depend on the diameters of the bile duct and stone, and that it has higher detectability than other modalities [16,19]. However, EUS is more invasive than ultrasonography, CT, or MRCP. Even if EUS has been completed in a patient with severe pancreatitis to detect stones, careful consideration is necessary as to whether we can quickly move on to ERCP or EST. Treatment was successful in all patients and no patient had pancreatitis progressed. Bile duct cannulation was easily successful in many cases probably because many patients had decreased function of the sphincter of Oddi due to the spread of inflammation. This suggests the reason why pancreatitis has not aggravated. This time, there was no case of difficult insertion into the bile duct. It is presumable, however, that there may be some cases of difficult insertion into the bile duct and some cases of cholangiogram inevitably performed. Cases of difficult insertion into the bile duct and cholangiogram are risk factors for post-ERCP pancreatitis [20], potentially aggravating the disease condition. In such cases, pancreatic duct stenting is performed to avoid elevation of intraductal pressure of the pancreas as much as possible, and measures, such as securing the outflow tract of pancreatic juice, should be considered to avoid aggravation of pancreatitis [20-22]. If gallstone pancreatitis develops, cholecystectomy is recommended after pancreatitis becomes quiescent [1]. In this study, 66.7% (72/108) did not undergo cholecystectomy owing to the patient background or upon request from the patient. However, no patient had acute pancreatitis relapsed during the three-year course of observation. No relapse was considered as a result of EST, which was performed in a way to make the opening of the bile duct separate from that of the pancreatic duct, preventing the pancreatic duct from being obstructed. However, cholangitis attributed to a fall of gallbladder stone was noted in three patients. Whether relapse of pancreatitis can be prevented with EST is debatable as insufficient incision in EST may result in relapse, and thus, careful consideration is still needed. In this study, relapse rates of pancreatitis and hepatobiliary complications were low. As a conclusion, we found that cholecystectomy was not necessarily required after calming down gallstone pancreatitis. However, the comparison of the group treated with ERCP+EST alone and the group treated with cholecystectomy in three randomized controlled trials in the past revealed that a significantly higher number of hepatobiliary complications were reported in ERCP+EST alone group [23-25]. Therapeutic choices must be made with due consideration of the patient background and the informed consent. Although the sample size is large in this study on ERCP and EST relating to gallstone pancreatitis, this is a retrospective study. Hereafter, a prospective study will be needed for further investigation.

## Conclusions

It was suggested that ERCP and EST could be useful therapies for gallstone pancreatitis. Gallstone pancreatitis is a disease with a poor prognosis if it becomes severe. Once a diagnosis of gallstone pancreatitis is made, ERCP-related procedures should be considered promptly. However, caution is required as ERCP and EST may worsen pancreatitis, and these procedures should be performed by experts.
